# Organ Specificity and Heterogeneity of Cancer-Associated Fibroblasts in Colorectal Cancer

**DOI:** 10.3390/ijms222010973

**Published:** 2021-10-11

**Authors:** Naoya Miyashita, Akira Saito

**Affiliations:** Department of Respiratory Medicine, Graduate School of Medicine, University of Tokyo, Tokyo 113-0033, Japan

**Keywords:** cancer-associated fibroblast, colorectal cancer, TGF-β, PDGF, FOXL1

## Abstract

Fibroblasts constitute a ubiquitous mesenchymal cell type and produce the extracellular matrix (ECM) of connective tissue, thereby providing the structural basis of various organs. Fibroblasts display differential transcriptional patterns unique to the organ of their origin and they can be activated by common stimuli such as transforming growth factor-β (TGF-β) and platelet-derived growth factor (PDGF) signaling. Cancer-associated fibroblasts (CAFs) reside in the cancer tissue and contribute to cancer progression by influencing cancer cell growth, invasion, angiogenesis and tumor immunity. CAFs impact on the tumor microenvironment by remodeling the ECM and secreting soluble factors such as chemokines and growth factors. Differential expression patterns of molecular markers suggest heterogeneous features of CAFs in terms of their function, pathogenic role and cellular origin. Recent studies elucidated the bimodal action of CAFs on cancer progression and suggest a subgroup of CAFs with tumor-suppressive effects. This review attempts to describe cellular features of colorectal CAFs with an emphasis on their heterogeneity and functional diversity.

## 1. Cancer-Associated Fibroblast as a Key Component of Cancer Tissue

Fibroblasts are ubiquitous mesenchymal cells that regulate synthesis, degradation and turnover of extracellular matrix (ECM) in the connective tissue, thus providing structural scaffolds for a variety of cell types [1,2]. Fibroblasts not only support the tissue architecture but also modulate intercellular communications by producing soluble factors, including cytokines, chemokines and growth factors. During the process of wound healing, fibroblasts are essential for tissue regeneration, and dysregulation of the fibroblast-mediated repair process is related to scar formation and fibrotic diseases [3]. For example, fibroblasts are key effector cells in the pathogenesis of pulmonary fibrosis, renal fibrosis, scleroderma, liver cirrhosis and tissue fibrosis of the gastrointestinal tract.

Primary cultures of fibroblasts could be established easily compared to those of epithelial cells, and fibroblasts have been widely exploited for experimental cellular models [4,5]. Recent studies have revealed multiple layers of fibroblast heterogeneity. Gene expression profiles of fibroblasts vary depending on the anatomical site of their origin [6]. During organogenesis of various organs, reciprocal interactions between epithelial and mesenchymal cells are essential. It has been described that the cellular property and differentiation of epithelial cells are properly regulated when cultured with fibroblasts derived from the same organ. For instance, clonal growth and differentiation of alveolar epithelial cells can be achieved when co-cultured with lung fibroblasts [7]. For another example, the efficiency of colonoid formation from isolated crypts is enhanced by a co-culture system using colonic fibroblasts [8]. Thus, fibroblasts seem to have organ-specific features and play differential roles according to the anatomical location.

Cancer can be conceived as a new organ that comprises tumor cells and the surrounding stroma that co-evolve during cancer progression [9]. A tumor stroma consists of tumor vasculature, various immune cells (such as T cells, B cells, macrophages and granulocytes), fibroblasts and ECM. The pathological importance of tumor stroma has been shown by the observations that stromal gene expression profiles are predictive of patient prognosis of various cancer types [10,11,12].

In recent decades, cancer therapeutics targeting tumor angiogenesis have made remarkable progress and are clinically in use [13]. Furthermore, marked success in immune checkpoint blockade for cancer treatment strongly supports the concept that modifications in tumor stroma can effectively control cancer progression. Cancer-associated fibroblasts (CAFs) are major constituents of the tumor stroma [14,15], and a number of studies have demonstrated that CAFs support the tumor microenvironment, which fuels the growth and spread of cancer cells. Mechanistically, CAFs play significant roles in invasion, angiogenesis, cancer cell stemness and drug resistance [16,17]. Therefore, CAFs might be a promising target for next-generation cancer therapeutics.

Colorectal cancer (CRC) is the third most common cancer and accounts for approximately 10% of cancers worldwide. Despite recent advances in chemotherapy and molecular-targeted therapies [18], CRC is the second most common cause of cancer-related mortality [19]. Importantly, its incidence is increasing presumably due to the influence of dietary habits and obesity. Comprehensive cancer genome profiling has revealed molecular changes in CRC, including high microsatellite instability, copy number alterations and gene mutations such as *APC*, *TP53*, *SMAD4*, *PIK3CA* and *KRAS* [20,21]. In parallel, accumulating evidence suggests that activation of colorectal CAFs is associated with metastasis, therapeutic response and patient prognosis, underscoring their critical role in CRC progression [10,22,23]. Transforming growth factor (TGF-β) signaling activation of tumor stroma predicts disease relapse in CRC, and TGF-β target gene expression in CAFs is implicated in metastasis initiation by CRC cells [23]. Expression levels of gene sets associated with poor prognosis in CRC are prominently elevated in CAFs, followed by endothelial cells and leukocytes [10]. Moreover, high expression levels of genes upregulated in CAFs are indicative of a poor prognosis for CRC patients [10]. In this review, we discuss the phenotypic and functional diversity of CAFs, with an emphasis on their pathological role in CRC.

## 2. Diversity and Organ Specificity of Fibroblasts

Fibroblasts reside in the ECM and are involved in ECM turnover and tissue homeostasis. Fibroblasts cultured on plastic culture dishes show a spindle-like appearance and it is difficult to distinguish the organ of their origin based on cell morphology. However, transcriptome analyses in previous studies have demonstrated that fibroblasts obtained from different organs show unique gene expression patterns [6,24].

A pioneering study that performed microarray analyses on 50 fibroblasts derived from different anatomical sites revealed that cultured fibroblasts have positional memory characterized by gene expression patterns [6]. Furthermore, this study demonstrated that fibroblasts from each topographic site display unique patterns of HOX family genes and the fibroblasts could be grouped together based solely on the HOX gene expression profiles [6].

Additionally, the gene expression patterns of 47 primary fibroblast populations from 43 unique anatomical sites could be categorized into three subtypes related to characteristic anatomical divisions: anterior–posterior, proximal–distal and dermal–nondermal [24]. This study also identified the site-specific expression of HOX genes in adult fibroblasts. For example, HOXA13 was selectively expressed in fibroblasts from developmentally distal structures, such as fingers, feet and foreskin, but not in fibroblasts more proximal in the arm, leg and trunk [24].

Another interesting study performed gene expression profiling of 63 fibroblasts from 13 organs and demonstrated that gastrointestinal fibroblasts (GIFs) display unique gene signatures distinct from non-GIFs [25]. Furthermore, transcriptomic features of GIFs were different depending on their anatomical sites, including the esophagus, stomach, duodenum, ileum and colon [25].

More recently, an integrative analyses of 28 single-cell RNA-sequencing (RNA-seq) datasets across 16 tissues successfully constructed a fibroblast-specific single-cell atlas [26]. This study identified universal fibroblast subtypes marked by Pi16 or Col15a1 expression that are ubiquitously present in nearly all tissues. Transcriptomic studies suggested that these universal fibroblasts can differentiate into activated fibroblasts in the disease state. Importantly, transcriptional states of universal and activated fibroblasts were found to be conserved between mice and humans [26]. These findings suggest that fibroblasts with tissue-specific features may commonly arise from universal fibroblasts.

We previously analyzed gene expression profiling data of 45 fibroblasts from different anatomical sites available from the FANTOM5 dataset [27], and identified lung- or gingiva-specific gene signatures of fibroblasts [28,29]. Of pathological importance, we found that the genes with higher expression in lung fibroblasts relative to other fibroblasts were globally downregulated in lung CAFs [29]. Thus, loss of organ-specific fibroblast gene signatures is associated with the acquisition of a CAF phenotype.

**Figure 1:**
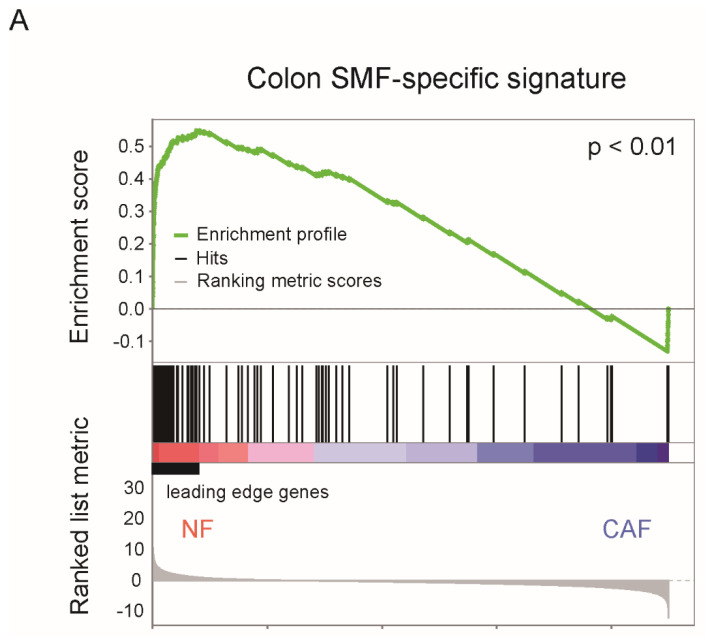

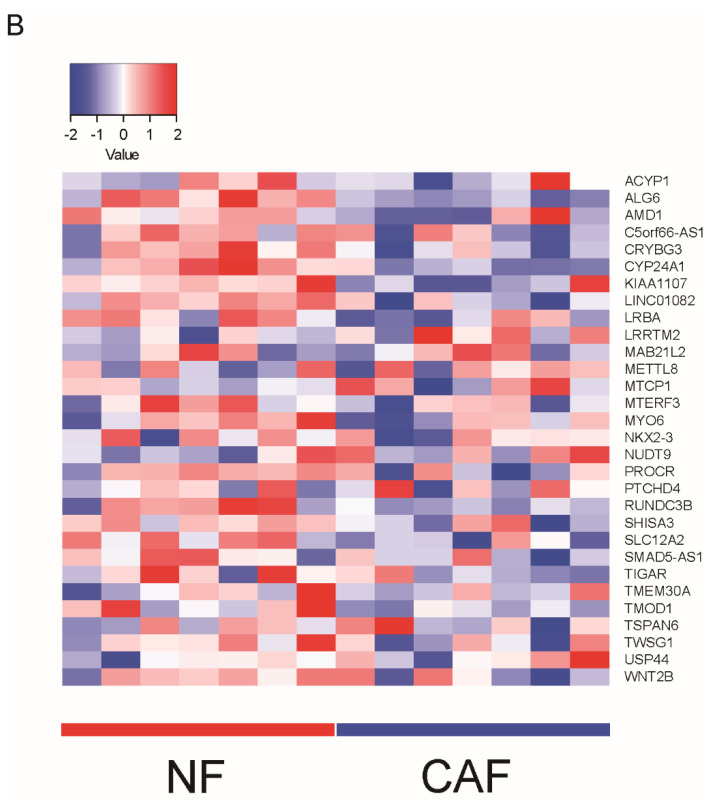
Unique features of colon submucosal fibroblasts. (**A**) Gene set enrichment analysis (GSEA) was performed using the microarray datasets of colon normal fibroblasts (NFs) and cancer-associated fibroblasts (CAFs). Expression profiling data of colon NFs and colorectal CAFs were obtained from the GSE70468 dataset. A colon SMF-specific signature was identified by differential expression analysis between the colon SMFs (4 samples) and non-GIFs (31samples) obtained from the GSE63626 dataset. SMFs: submucosal fibroblasts. GIFs: gastrointestinal fibroblasts. (**B**) Heatmap of the relative expression levels of 30 leading-edge genes obtained by GSEA.

Similarly, we performed an integrative analysis on the publicly available transcriptome data of colon fibroblasts (GSE63626) and colorectal CAFs (GSE70468), and found that the genes with higher expression in colon submucosal fibroblasts relative to non-GIFs were downregulated in colorectal CAFs compared with normal fibroblasts (NFs) (Figure 1A) [25,30]. Interestingly, the 30 leading-edge genes that showed high enrichment in NFs and downregulation in colorectal CAFs included NK2 homeobox 3 (NKX2-3) and Wnt family member 2B (WNT2B). Thus, loss of these genes unique to colon submucosal fibroblasts may be related to the development of colorectal CAFs (Figure 1B).

**Figure 2:**
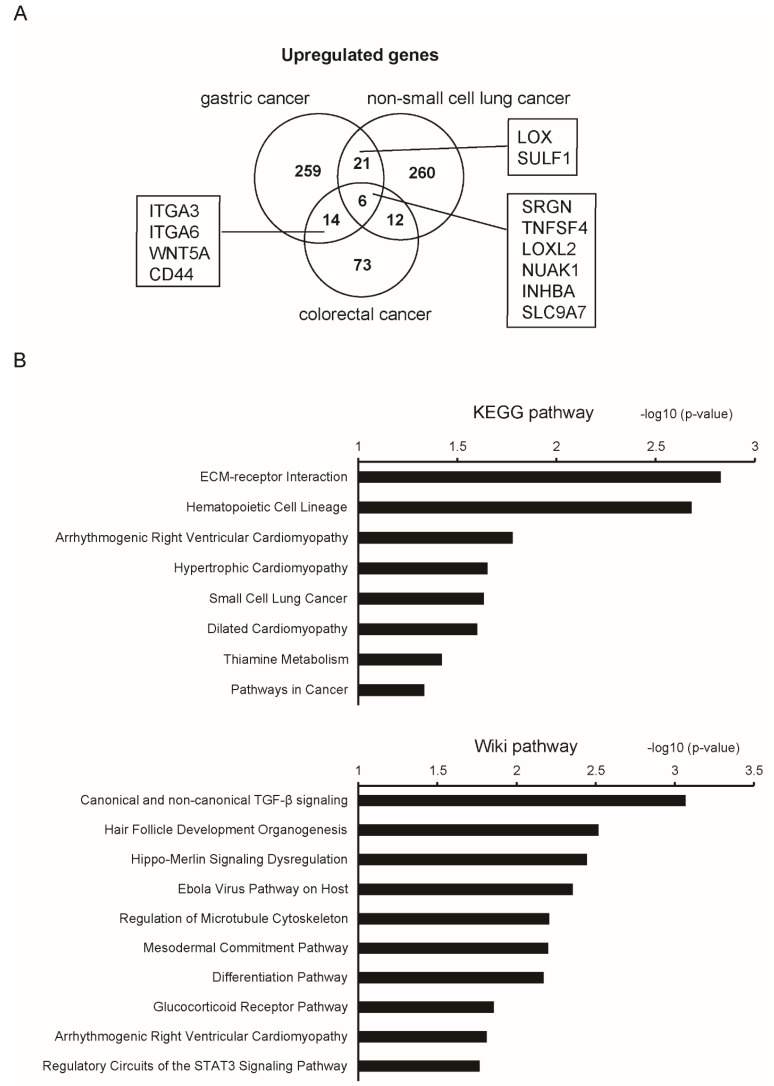
Upregulated genes in CAFs identified in different cancer types. (**A**) Venn diagram showing the genes upregulated in CAFs relative to NFs in colorectal cancer (CRC), gastric cancer and non-small cell lung cancer. The number of identified genes is indicated. Representative overlapping genes are indicated. Upregulated genes in CAFs were identified as follows. CRC: expression profiling data by microarray were obtained from the GSE70468 dataset. Upregulated genes in CAFs relative to their normal counterparts were extracted by the thresholds of a *p*-value < 0.05 and log fold-change > 1. Gastric cancer: fragments per kilobase of exon per million reads mapped (FPKM) data of 11 matched NFs and CAFs were obtained from the GSE83834 dataset. Deseq2 was performed and the top 300 upregulated genes were extracted. Non-small cell lung cancer: expression profiling data by microarray were obtained from the GSE22874 dataset. Upregulated genes in CAFs relative to NFs were extracted by the thresholds of a *p*-value < 0.05 and log fold-change > 1. (**B**) Pathway analyses (KEGG and Wiki pathways) were performed using CAF-upregulated genes in at least two cancer types.

In the last decade, several studies performed global gene expression profiling on isolated CAFs and patient-matched NFs derived from the same organs [12,30,31,32]. We compared the dysregulated genes in CAFs relative to their normal counterparts identified in different cancer types (CRC, gastric cancer and non-small cell lung cancer), and found that such gene signatures are largely overlapping among the different organs (Figure 2A). It was noteworthy that the overlapping genes included those related to ECM remodeling and cell–ECM interactions; for example, lysyl oxidase (LOX), lysyl oxidase like 2 (LOXL2), sulfatase 1 (SULF1), integrin subunit alpha 3 (ITGA3), ITGA6 and CD44. Pathway analyses using upregulated genes by at least two cancer types demonstrated that these CAF signatures are closely related to TGF-β signaling activation (Figure 2B) [12,30,31,32]. This similarity was in contrast to the organ-dependent transcriptomic difference of normal fibroblasts.

Recently, genome-wide DNA methylation and gene expression profiling of lung CAFs and patient-matched NFs identified a methylation index of 54 CpG sites for CAF/NF discrimination [33]. Of clinical importance, this methylation index could stratify the malignant status of the tumor microenvironment among different patients and serve as a biomarker to predict recurrence in multiple lung cancer cohorts.

Further studies on epigenetic alterations (DNA methylation, histone modification and chromatin remodeling) using novel techniques such as ATAC-sequencing would provide deeper insights into CAF heterogeneity.

## 3. Phenotypic Heterogeneity of CAFs

CAFs reside in the cancer tissue and are morphologically identified as spindle-shaped cells distinct from immune cells, endothelial cells and cancer cells [34]. To date, several molecular markers for fibroblasts or CAFs have been proposed. Vimentin and S100A4 (also known as fibroblast-specific protein 1, FSP1) are considered to be markers for fibroblasts. However, they are not highly specific and are expressed in other mesenchymal cells or hematopoietic cells [35]. Therefore, it is necessary to analyze cell marker expression together with morphological evaluation for the identification of fibroblasts.

Fibroblasts can be activated by several soluble factors, including TGF-β and platelet-derived growth factor (PDGF) [14,36]. TGF-β potently stimulates the production of α-smooth muscle actin (α-SMA, also known as ACTA2) and stress fiber formation. These activated fibroblasts positive for α-SMA are highly capable of ECM production and termed as myofibroblasts [37]. Numerous studies have used α-SMA as a marker for CAFs.

It has been reported that TGF-β staining in CRC tissue was strongly enhanced in the tumor stroma, and most fibroblasts showed the myofibroblast phenotype positive for α-SMA [38]. Cell culture experiments demonstrated that TGF-β1 stimulation of CAFs led to a strong increase in TGF-β1 protein production and promoted invasion of CAFs into the collagen matrix. Furthermore, most of the CAFs treated with the conditioned medium of CRC cells were positive for α-SMA and showed hyperactivated TGF-β signaling accompanied by increased expression of TGF-β target genes such as matrix metalloproteinases (MMPs). These observations indicated that interactions between epithelial tumor cells and CAFs amplifies TGF-β signaling and increases proteinase expression, thereby creating the tumor-promoting microenvironment [38].

PDGF signaling enhances proliferation and migration of fibroblasts, acting as a key determinant of fibroblast function [39]. It is thought that fibroblasts activated by PDGF acquire pro-tumorigenic properties and a previous study showed that the migration and invasion of CRC cells were enhanced when co-cultured with PDGF-stimulated fibroblasts—at least partly through the effect of the staniocalcin 1 (STC1) expressed by the fibroblasts [40].

In addition to α-SMA, previous reports described several CAF markers with prognostic and functional relevance, including caveolin-1 (CAV1) [41], CD90 (also known as Thy-1) [42] and fibroblast activation protein (FAP) [43]. Importantly, it has been shown that high expression levels of α-SMA and FAP are associated with poor disease-free or overall survival of CRC patients [43].

CAV1 is involved in mechanotransduction and ECM remodeling by regulating Rho GTPase activity. CAV1-expressing fibroblasts are abundant in the tumor stroma of multiple cancer types, including CRC, and stromal CAV1 expression is associated with a poor prognosis [41]. IL-6 expression is increased in CRC tissue and tumor stromal fibroblasts expressing CD90 are the major cell phenotype producing IL-6 [42]. CD90-positive CAFs directly support the growth of stem-like populations within CRC cell lines and also modulate tumor immune responses that favor CRC progression [42].

These CAF markers show differential expression patterns depending on the analyzed cancer type and disease stage, supporting the idea that various subpopulations of CAFs exist in the cancer tissue.

Fibroblast heterogeneity can be partly explained by variable activation levels of the resident fibroblasts with organ-specific features. The cellular origin of CAFs is also heterogeneous and previous studies described that bone marrow-derived precursors or tissue-resident mesenchymal stromal cells can be a cellular source of CAFs [44,45]. Of particular note, it has been demonstrated that mesenchymal stem cells convert into CAFs when injected into tumor-bearing mice [46].

In recent years, intra-tumoral heterogeneity of stromal cells or CAFs have been dissected in detail by single-cell transcriptomics. Single-cell RNA-seq analyses of 52,698 cells from five lung cancer tissues identified five distinct subtypes of fibroblasts that express different types of collagens [47]. One cluster showed higher expression of genes related to TGF-β signaling, ECM and epithelial–mesenchymal transition (EMT); for example, MMP2 and collagen type Iα2 (COL1A2). Another cluster showed higher expression of ACTA2 and myogenesis-related genes, such as myosin heavy chain 11 (MYH11) and myocyte enhancer factor 2C (MEF2C).

Single-cell RNA-seq analyses of 969 cells from 11 CRC samples revealed two subtypes of CAFs [48]. One cluster showed higher expression of ECM-related genes whereas the other expressed myofibroblast-related genes such as ACTA2 and transgelin (TAGLN). These findings at single-cell resolution will be further reinforced in future studies by using more recently developed technologies, such as spatial transcriptomics [49].

## 4. Functional Diversity of Fibroblasts

It has been experimentally demonstrated that inoculation of fibroblasts enhances tumorigenesis of xenografted cancer cells [50,51]. In animal models, CAFs stimulate tumor cell proliferation, promote angiogenesis and modulate immune cell infiltration.

It is also known that close interactions with fibroblasts facilitate cancer cell invasion [17]. Collective invasion of co-cultured cancer cells and stromal fibroblasts revealed that fibroblasts serve as leading cells and facilitate the movement of cancer cells within tracks in the ECM behind fibroblasts. Protease- and force-mediated ECM remodeling is required for the generation of these tracks by fibroblasts. Integrin signaling that mediates cell–ECM interactions is involved in the mechanism of collective invasion [17].

CAFs directly stimulate cancer cell growth and also enhance tumor angiogenesis by paracrine signaling, such as vascular endothelial growth factor (VEGF) [52], hepatocyte growth factor (HGF) [53], growth differentiation factor 15 (GDF15) [54], C-X-C Motif Chemokine Ligand 12 (CXCL12) [55] and CXCL14 [51].

VEGF and HGF are known as pro-angiogenic factors and bevacizumab, a humanized anti-VEGF monoclonal antibody, is used for the treatment of CRC [13]. Myofibroblasts isolated from the CRC tissue or fibroblasts stimulated by TGF-β are capable of enhancing CRC cell invasion and this effect is mediated by HGF and tenascin C (TNC) [53]. GDF15 is highly expressed in prostate CAFs and fibroblast-derived GDF15 exerts paracrine effects on cancer cell migration, invasion and tumor growth [54].

CXCL12 directly stimulates tumor growth and also promotes angiogenesis by recruiting bone marrow-derived endothelial progenitor cells [55]. CXCL14 is upregulated in prostate CAFs and co-injected CXCL14-expressing fibroblasts promote xenografted tumor growth, angiogenesis and macrophage infiltration [51].

In addition to the secretion of soluble factors, CAFs remodel the tumor stroma by producing the ECM components, including collagen, proteoglycan, laminin and hyaluronan, as well as proteases, such as MMPs. TGF-β potently elicits the expression of the ECM components, integrins, MMPs and protease inhibitors [56].

It has been shown that CAF-mediated tissue contraction and matrix stiffening lead to cell stretching, which activates Yes1 associated transcriptional regulator (YAP) signaling in CAFs. Such mechanotransduction and YAP activation further augment CAF features, forming a feed-forward loop during tumor progression [57]. Therefore, mechanical stress at the levels of ECM stiffness and cellular tension seems to be generated, sensed, modulated and sustained by the crosstalk between TGF-β and YAP [56].

CAFs derived from different CRC patients show variable cellular responses or gene expression patterns. Herrera et al. established primary CAFs and NFs from 15 human CRC samples and found differences in the pro-migratory effects on CRC cells [4]. Moreover, they demonstrated that a gene signature of pro-tumorigenic CAFs could predict a poor prognosis for CRC patients.

Another study analyzed eight pairs of colorectal CAFs and NFs, and identified a 108-gene signature differentially expressed in CAFs relative to NFs [32]. Higher scores of this signature were related to higher proliferative and migratory properties. This study also showed that CAFs were more potent in enhancing cancer cell migration and tumor formation. Importantly, approximately half of the genes dysregulated in CAFs were specifically expressed in fibroblasts as compared to epithelial, endothelial or hematopoietic cells [32].

Contrary to the prevailing view of CAFs as a cell type that promotes tumor progression, accumulating evidence shows that subsets of CAFs have tumor-suppressive functions [58,59]. It has been described that Meflin, a glycosylphosphatidyl inositol (GPI)-anchored protein is a marker for cancer-restraining CAFs in pancreatic ductal adenocarcinoma (PDAC) [60,61]. Interestingly, Meflin is also expressed in undifferentiated mesenchymal stem/stromal cells and is downregulated by TGF-β signaling or substrate stiffness, suggesting that loss of Meflin expression is associated with phenotypic induction of tumor-promoting CAFs [62].

In genetically engineered mouse models that develop spontaneous PDAC, specific deletion of Col1A1 in αSMA-positive myofibroblasts accelerated cancer progression [63]. Meanwhile, Col1A1 deletion in Fsp1-positive stromal cells did not affect collagen deposition or disease progression. Col1A1 deletion in αSMA-positive myofibroblasts led to alterations in immune responses: increased myeloid-derived suppressor cells (MDSCs) and decreased lymphocytes. Cxcl5, a potent MDSC chemoattractant, was expressed in cancer cells, and its production was enhanced by decreased type I collagen in the tumor stroma [63]. Thus, collagen production by CAFs may have a tumor-restricting aspect by modulating the tumor immune landscape.

Recently, both tumor-promoting and tumor-suppressive functions in different CAF subtypes have been investigated in more detail. In mouse models of desmoplastic liver metastasis, hepatic stellate cell-derived CAFs segregated into myofibroblastic CAF (myCAF), inflammatory CAF (iCAF) and mesothelial CAF (mesCAF) based on single-cell RNA-seq analyses [64]. CAF-selective deletion of type I collagen resulted in significantly increased metastatic tumor growth, indicating the tumor-restricting effect of type I collagen that overrides the matrix stiffness-mediated pro-tumorigenic signals. On the other hand, myCAF-secreted hyaluronan and iCAF-secreted HGF exerted tumor-promoting effects. Noteworthy, both hyaluronan synthase 2 (Has2) and Col1a1 were mainly expressed in myCAF, suggesting that myCAF has opposing roles depending on the cellular context [64].

Forkhead box L1 (FOXL1) is an evolutionarily conserved transcription factor expressed in the mesenchyme surrounding the anterior gut during organogenesis. Foxl1-deficient mice show defects in their gastrointestinal tracts [65], and Foxl1-positive subepithelial fibroblasts constitute the intestinal stem cell niche [66]. Epithelial cell differentiation and regeneration are controlled by BMP signaling in the alveolus [67] and we demonstrated that FOXL1 regulates the expression of the BMP ligands (BMP2 and BMP4) and BMP antagonists, including gremlin 1 (GREM1) in lung fibroblasts [68]. Considering the recent report showing that BMP signaling inhibition by GREM1 contributes to CRC progression [69], FOXL1 expression in the intestinal stem cell niche or subepithelial fibroblasts may be related to colorectal carcinogenesis.

## 5. CAFs Modulate Tumor Immunity

Immune checkpoint inhibitors (ICIs) have become a standard therapeutic option for multiple cancer types, including microsatellite instability-high CRC [70], and the pathological impact of tumor immunity on cancer progression has been increasingly appreciated in recent years. In a mouse model, depletion of FAP-positive fibroblasts resulted in cytokine-induced hypoxic necrosis of tumor cells, highlighting the critical role of fibroblasts in tumor immune suppression [71].

A recent report demonstrated that LRRC15-positive CAFs are predictive of poor therapeutic response to ICIs, suggesting that a subset of CAFs critically participate in tumor immunity [72]. Thus, interactions between CAFs and immune cells draw increasing attention as a key determinant of tumor progression, and growing evidence indicates that CAFs regulate recruitment, activation and removal of immune cells in the tumor microenvironment [73].

CAFs induce depletion and dysfunction of tumor-specific T cells by antigen cross-presentation and cellular interactions mediated by Fas ligand (FASL) and programmed cell death 1 ligand 2 (PD-L2) [74]. A previous study showed that CXCL12 secreted by CAFs limits T cell recruitment into the cancer tissue, and CAF depletion or CXCL12 inhibition leads to T cell accumulation, thereby enhancing the therapeutic effect of ICI in a mouse PDAC model [75]. Another report showed that CXCL12 secreted by a subset of CAFs recruits CD4+CD25+ T cells and promotes their differentiation into regulatory T cells, thereby suppressing antitumor immunity in human breast cancer [76].

Tumor-associated macrophages (TAMs) are polarized to the M2 phenotype, and they produce immunosuppressive cytokines such as TGF-β and IL-10 [77,78]. M2 macrophages further induce immune suppression and promote tumor progression through TGF-β-mediated activation of CAFs [73,79]. Conversely, CAFs stimulate TAMs or MDSCs via secretion of C3a, C-C motif chemokine ligand 2 (CCL2) and chitinase 3-like protein 1 (CHI3L1, also known as YKL-40) [80,81,82,83,84,85].

C3a is a cleaved product of the complement component C3 and supports the recruitment of C3aR-expressing macrophages. Single-cell RNA-seq analyses of the stromal compartment in murine melanoma revealed that C3 is specifically upregulated in the CD34-high CAF subpopulation, suggesting its potential usefulness as a biomarker [80].

In a murine liver tumor, FAP is responsible for the inflammatory phenotype of CAFs via activation of the JAK2–STAT3 pathway, and FAP-positive CAFs are a major source of CCL2 [81]. In this model, CAF-derived CCL2 signaling promoted tumor growth by enhancing the recruitment of MDSCs and supporting cancer immune evasion. Another report using a CRC mouse model also demonstrated that FAP-high CAFs promote immunosuppression via CCL2 production [82]. Mechanistically, CCL2 recruited myeloid cells and decreased T cell activity. In human CRC tissue samples, FAP expression was associated with myeloid cell infiltration and was inversely related to T cell number [82].

CAF-derived CHI3L1 drives an immunosuppressive microenvironment in breast tumors. ChI3L1 is highly expressed in CAFs isolated from mouse mammary tumors and in the stroma of human breast carcinoma. ChI3L1 silencing in fibroblasts attenuated tumor growth, macrophage recruitment and its polarization to the M2 phenotype while enhancing T cell infiltration and the Th1 response. Thus, fibroblast-derived ChI3L1 facilitates tumor progression by remodeling the tumor immune microenvironment in breast cancer [83].

In prostate carcinoma, both CAFs and cancer cells enhance cell migration of monocytes and promote their transdifferentiation toward M2 macrophages, which in turn activates CAFs [84]. This reciprocal interactions between CAFs and M2 macrophages contribute to enhanced tumor cell motility. Moreover, M2 macrophages stimulated by CAFs and cancer cells display pro-angiogenic effects and their accumulation in the tumor tissue correlates with prostate cancer aggressiveness [84].

Monocytes are thought to be the precursors of TAMs and a previous study tested monocyte migration into cell spheroids [85]. Monocyte infiltration toward tumor-derived fibroblast spheroids was high and considerable amounts of CCL2 were secreted by fibroblast spheroids. Blockade of CCL2-CCR2A/2B signaling inhibited monocyte migration, suggesting the importance of the CCL2-mediated mechanism for monocyte infiltration into the tumor stroma [85].

## 6. Future Perspectives

CRC shows inter-patient and intra-tumor heterogeneity [86,87]. The CRC Subtyping Consortium (CRCSC) identified four consensus molecular subtypes (CMS1 to CMS4) and characterized their biological features by integrating the available data of their transcriptome, microsatellite instability, mutation profiles, DNA methylation and somatic copy number alterations [88]. Interestingly, CMS4 (mesenchymal subtype) exhibited a gene expression profile indicating TGF-β activation and overexpression of ECM proteins, and displayed worse overall survival and relapse-free survival. Given that TGF-β signaling and ECM deposition are closely associated with CAF activation, therapeutic approaches targeting CAFs may be beneficial for this subset of CRC patients.

Most recently, pan-cancer transcriptome analyses revealed four conserved subtypes of the tumor microenvironment that correlate with response to immunotherapy [89]: immune-enriched, fibrotic (IE/F); immune-enriched, non-fibrotic (IE); fibrotic (F); and immune-depleted (D). Higher expression of genes related to angiogenesis, fibroblast and matrix remodeling was found in the IE/F and F subtypes and was associated with TGF-β signaling activation. Interestingly, tumor mutation burden was relatively low in the IE/F and F subtypes whereas T cell infiltration was high in the IE/F subtype but was low in the F subtype. The F subtype showed inferior prognosis across various cancer types and the worst response to ICIs, indicating the need for novel cancer therapeutics targeting fibroblasts and tissue fibrosis [89].

Further clarification of CAF heterogeneity and its molecular features would provide a deeper insight into CRC pathogenesis and may pave the way for the development of novel treatments for CRC.
